# Psychometric properties and cross-cultural adaptation of the Chinese version of the family caregiver communication tool-chronic illness

**DOI:** 10.3389/fpubh.2026.1865347

**Published:** 2026-08-11

**Authors:** Yingying Wang, Jiaxin Li, Yameng Shang, Yanmei Zhu, Fangli Liu

**Affiliations:** 1 School of Medicine, Henan Kaifeng College of Science Technology and Communication, Kaifeng, Henan, China; 2 School of Nursing, Xinyang Vocational and Technical College, Xinyang, Henan, China; 3 Department of Respiratory and Critical Care Medicine, The First Affiliated Hospital of Zhengzhou University, Zhengzhou, Henan, China; 4 Department of Nursing, The Seventh Affiliated Hospital, Sun Yat-sen University, Shenzhen, Guangdong, China; 5 School of Nursing and Health, Henan University, Kaifeng, Henan, China

**Keywords:** chronic diseases, family caregiver, family communication pattern, reliability, validity

## Abstract

**Background:**

With the rapid aging of the Chinese population, family caregivers play a pivotal role in the management of chronic diseases. Effective family communication is crucial for reducing caregiver burden and improving care quality. However, China lacks a generic, culturally adapted instrument to assess communication patterns in these specific settings. This study aimed to translate the Family Caregiver Communication Tool-Chronic Illness (FCCT-CI) into Chinese and evaluate its psychometric properties.

**Methods:**

Following Brislin’s translation model, the FCCT-CI was translated, back-translated, and cross-culturally adapted through expert consultation and pilot testing. From March to July 2023, a cross-sectional study was conducted with 319 family caregivers recruited via convenience sampling from a tertiary hospital in Henan Province. To ensure rigorous validation, the total sample was randomly split into two independent subsamples: Sample A (*n* = 110) was used for Exploratory Factor Analysis (EFA), and Sample B (*n* = 209) was used for Confirmatory Factor Analysis (CFA). Psychometric properties were evaluated using item analysis, content validity, construct validity, internal consistency, and test–retest reliability.

**Results:**

The Chinese FCCT-CI comprises 10 items across two dimensions. Exploratory Factor Analysis (Sample A) extracted two factors explaining 71.46% of the total variance. Confirmatory Factor Analysis (Sample B) demonstrated excellent model fit (*χ*^2^/*df* = 1.707, CFI = 0.988, TLI = 0.979, RMSEA = 0.058). Content validity was robust, with an S-CVI/Ave of 0.94 and I-CVI ranging from 0.80 to 1.00. The scale exhibited high reliability, with a Cronbach’s *α* of 0.846, split-half reliability of 0.943, and a test–retest intraclass correlation coefficient (ICC) of 0.920.

**Conclusion:**

The Chinese version of the FCCT-CI demonstrates good reliability and validity. It provides a reliable tool for assessing family communication patterns among family caregivers of patients with chronic diseases in China.

## 1 Introduction

Non-communicable diseases (NCDs) constitute a leading global health burden, with their pathogenesis closely associated with genetic, physiological, environmental, and behavioral determinants (1). China confronts a particularly acute challenge arising from its rapidly aging population. By the end of 2022, individuals aged 60 years or older numbered 280 million, representing 19.8% of the total population (2). This demographic shift is paralleled by a high prevalence of chronic illness; the rate among Chinese older adults reaches 71.8%, with NCDs accounting for 88.5% of all-cause mortality (3). Such conditions frequently result in prolonged functional impairment and a profound dependence on external care. While national policy promotes integrated prevention and management alongside ageing in place (4), the family unit remains the predominant care setting due to constraints in formal care service provision and the enduring influence of socio-cultural norms (5). Family members who assume primary caregiving roles commonly experience substantial psychological and physical strain while managing demanding and sustained care responsibilities (6, 7). Their challenges in communication and perceived lack of sup-port thus warrant focused attention.

Among the myriad stressors encountered, family communication patterns have been identified as a central factor compounding caregiver burden (8). These patterns refer to the characteristic ways in which family members exchange information and emotions regarding the patient’s illness and care needs. Caregivers often face multiple communication difficulties, including struggles to comprehend patient needs, ineffective information sharing among fellow caregivers, and insufficient emotional support (8, 9). Such obstacles not only precipitate negative affective states like anxiety and depression in caregivers but may also exacerbate familial conflict, ultimately undermining care quality (10). For instance, recent evidence from China demonstrates that heavy caregiver burden and associated self-stigma can even drive patients to actively avoid health information, further compounding communication breakdowns (11). Communication challenges become particularly pronounced during the end-of-life phase, often intensified by cultural sensitivities surrounding death-related discourse, potentially leading to caregiver emotional suppression and hindering the expression of patient preferences (12, 13). Consequently, the systematic assessment of family communication patterns is fundamental to understanding caregiving challenges, identifying at-risk families, and enhancing overall care outcomes.

Existing instruments for assessing this domain each possess specific emphases and inherent limitations. Disease-specific tools, such as the Cancer Communication Assessment Tool for Patients and Families (CCAT-PF) (14), the Family Avoidance of Communication about Cancer Scale (FACC) (15), and the Experienced Communication in Dementia Questionnaire (ECD-C) (16), provide depth in evaluating particular conditions (e.g., cancer or dementia) or singular communication behaviors (e.g., avoidance). Their generalisability, however, is limited, rendering direct application to families managing more prevalent chronic diseases like hypertension or diabetes problematic. General instruments, exemplified by the Caregiver-Centered Communication Questionnaire (CCCQ) (17), primarily evaluate external communication dynamics between healthcare providers and caregivers, rather than focusing on interactions within the family system itself. Although these tools are valuable, they primarily focus on specific disease contexts (e.g., cancer) or patient provider interactions. Consequently, China lacks a generic, theoretically grounded instrument to assess family communication patterns among caregivers of older adults with various chronic diseases. In this context, the FCCT-CI developed by Wittenberg et al. (18), presents distinctive utility. Grounded in family communication theory, it classifies communication pat-terns along two key dimensions, conversation and conformity, offering a theoretical framework for developing tailored interventions (19). However, this instrument has not yet undergone validation or application within the Chinese cultural context.

Hence, a compelling need exists for the systematic linguistic and cultural adaptation of the FCCT-CI. Firstly, Chinese society, steeped in Confucian heritage and collectivist values, exhibits unique characteristics in family decision-making and communication styles (20); employing Western-developed tools directly risks significant cultural measurement bias. Secondly, the current absence of a validated, generalis able instrument capable of scientifically assessing intra-family communication patterns among chronic disease patients in China substantially impedes the development of targeted supportive interventions. To address this gap, this study aims to culturally adapt and translate the FCCT-CI into Chinese and to evaluate its psychometric properties, including reliability and validity, among family caregivers of older adults with chronic diseases in China. The objective is to furnish a scientifically rigorous and culturally pertinent assessment tool to inform future evidence-based strategies for family communication support and intervention.

## 2 Materials and methods

To ensure the quality of the adaptation, this research relied on the framework set by the International Test Commission (ITC). Consequently, both the translation and validation steps aligned with the second edition of these guidelines (21).

### 2.1 Adaptation process

After obtaining consent from the research team led by Professor Wittenberg, we followed Brislin’s forward-backward model to conduct the linguistic and cultural adaptation (22). In accordance with this model, forward translation was conducted by two independent bilingual translators whose native language is Chinese. Both translators possess high proficiency in English—one is an English teacher certified at the Professional English Level 8, and the other holds a doctoral degree in chronic disease care with overseas study experience. They independently translated the original FCCT-CI from English into Chinese, yielding two initial translated versions: FCCT-CI-A1 and FCCT-CI-A2. Subsequently, the researcher compared these two versions with the original scale, facilitated an in-depth discussion with both translators to address discrepancies in expression and meaning, and synthesized the results. Through this consensus process, a harmonized preliminary Chinese version, FCCT-CI-A3, was established.

The preliminary Chinese version (FCCT-CI-A3) was then independently back-translated into English by two other bilinguals who were blinded to the original FCCT-CI. The back-translation was performed by two individuals with doctoral qualifications in nursing and medicine, respectively, both of whom had studied abroad. This process produced two back-translated English versions: FCCT-CI-B1 and FCCT-CI-B2. A postgraduate nursing student compared and consolidated these two back-translations and organized a discussion between the back-translators to analyze and reconcile differences, resulting in a consolidated back-translated version, FCCT-CI-B3. To verify that the translated scale maintained semantic, conceptual, and content consistency with the original, the back-translated version and the translation process were reviewed by the original scale author for feedback on any ambiguous or contentious items.

A two-round Delphi expert panel review was conducted for cultural adaptation and content validity assessment. Ten experts from diverse fields—including clinical medicine, chronic disease nursing, geriatric care, nursing psychology, community nursing, rehabilitation medicine, medical humanities, and social medicine—were consulted. All experts held senior professional titles (professor or associate professor) and had a minimum of 10 years of experience in their respective fields. The expert panel evaluated each item for clarity, conceptual accuracy, cultural appropriateness, and relevance (using a four-point relevance scale: 1 = not relevant; 2 = weakly relevant; 3 = moderately relevant; 4 = highly relevant). Content validity was assessed using the Item-level Content Validity Index (I-CVI) and the Scale-level Content Validity Index (S-CVI/Ave), with acceptance criteria set at I-CVI ≥ 0.78 and S-CVI/Ave ≥ 0.90 (23). Expert authority was quantified using the authority coefficient (Cr), calculated as the mean of the judgment basis (Ca) and familiarity (Cs), with Cr ≥ 0.70 considered acceptable (24). Biographical information (age, education level, professional title, years of experience, and field of expertise) was also collected. The consultation materials were then distributed to the 10 experts via email and in-person meetings. All 10 experts provided their comments and suggestions. The research team discussed the revision suggestions and refined the translation accordingly, with consultation from the original author where necessary. After these procedures, a consensus was achieved and the final Chinese version of the FCCT-CI was formed for psychometric testing.

A pilot assessment involving 30 family caregivers from a hospital in Henan was undertaken to ensure the draft scale was easy to understand. Participants were recruited via convenience sampling and gave written consent after understanding the study procedures. The caregivers were then asked to complete the preliminary FCCT-CI. The time required for completion was recorded, and cognitive interviews were conducted to assess item comprehension. Items were considered problematic if more than 20% of participants rated them as “unclear” or requested clarification during cognitive interviews. The pilot test results showed that the average completion time was 8–10 min. All 30 participants reported that the scale instructions and item wording were clear and easy to understand. No items were identified as problematic based on the pre-established criterion. Based on this feedback, the research team refined the wording of several items to enhance clarity and cultural appropriateness while ensuring semantic equivalence with the source instrument, and the final Chinese version of the FCCT-CI was confirmed without further modifications to the item structure.

### 2.2 Psychometric evaluation of the FCCT-CI

#### 2.2.1 Participants and eligibility criteria

Using a cross-sectional approach, this study evaluated the psychometric performance (reliability and validity) of the translated FCCT-CI in Chinese family caregivers. We enrolled participants via convenience sampling from the Department of Geriatrics at a tertiary Class A hospital in Henan Province from March to July 2023.

Inclusion criteria: (1) Patient age ≥ 60 years; (2) Patients diagnosed with chronic diseases by secondary or higher-level medical institutions; (3) Patients partially or fully dependent on others for care (Barthel Index score < 100); (4) Family caregivers are family members or legal relatives of the patient, including spouses, children, etc.; (5) Care duration should be ≥ 3 months and ≥ 3 days per week (if there are multiple caregivers, the patient should designate the one with the longest care duration); (6) Family caregivers provide unpaid care; (7) Family caregivers should be at least 18 years old; (8) Family caregivers have normal cognitive and expressive abilities and can communicate effectively. Exclusion criteria: (1) Family caregivers with serious health impairments (e.g., severe heart, liver, or kidney failure, respiratory failure, or malignant tumors); (2) Family caregivers with a history of mental illness.

#### 2.2.2 Sample size and sampling strategy

Data collection was conducted in two phases using convenience sampling from March to July 2023. Phase 1 (March–April) targeted 110 participants for item analysis and Exploratory Factor Analysis (EFA), based on the recommendation of 10 subjects per item plus a 10% buffer for invalid responses (25). Phase 2 (May–July) targeted 220 participants for Confirmatory Factor Analysis (CFA) to satisfy the minimum requirement of 200 cases (23). In total, 319 valid questionnaires were obtained. Additionally, a random subsample of 30 caregivers was selected to assess test–retest reliability over a two-week interval.

#### 2.2.3 Data collection procedure

With the hospital’s permission, questionnaires were administered personally. The study goals and voluntary nature of participation were clarified beforehand. Once completed, the forms were collected on the spot. For participants who had difficulty reading, researchers read each item aloud and recorded their verbal responses to ensure data accuracy.

#### 2.2.4 Instruments

##### 2.2.4.1 Self-generated demographic characteristic questionnaire

The demographic information was collected using a researcher-developed questionnaire, which included the following variables: for older adult patients with chronic diseases, sex, age, marital status, education level, self-care ability, etc.; and for their family caregivers, sex, age, education level, relationship to the patient, duration of caregiving, etc.

##### 2.2.4.2 Barthel index

Developed by American scholar Dorothy Barthel (26), this scale is the most commonly used scale globally to assess activities of daily living (ADL). It was modified by Li et al. (27) and includes 10 items such as eating, dressing, and grooming. The scores range from 0 (complete dependence) to 100 (independence). Scores of 61–99 indicate mild dependence, 41–60 indicate moderate dependence, and ≤40 indicate severe dependence. A higher score indicates better daily living ability. The Cronbach’s *α* of the Chinese version of the scale is 0.916 (28).

##### 2.2.4.3 Chinese version of the FCCT-CI

The Chinese version of the FCCT-CI, after localization and cross-cultural adaptation, consists of 10 items divided into two dimensions: conversation and conformity. A 5-point Likert scale is used, with scores of 1–5 representing “never” to “frequently.” The two dimensions are scored separately, with each dimension ranging from 5 to 25 points. The type of family communication pattern is determined based on the scores of the two dimensions.

### 2.3 Statistical analysis

Statistical analyses were performed using SPSS 26.0 and AMOS 24.0. Categorical variables were summarized as frequencies and percentages, while continuous variables were presented as Mean ± Standard deviation ( $\bar{x}$ ± s).

#### 2.3.1 Item analysis

We evaluated item quality to improve the scale’s discrimination. This involved Pearson’s correlation analysis, where items with a coefficient (*r*) below 0.40 were eliminated. Additionally, we used the Critical Ratio (CR) method, comparing extreme groups (top vs. bottom 27%). Items with a CR ≥ 3.0 (*p* < 0.05) were retained as they demonstrated good discrimination (29).

#### 2.3.2 Reliability analysis

Internal consistency for the total scale and each dimension was measured via Cronbach’s *α*. The coefficient ≥ 0.70 was considered acceptable. Test–Retest Reliability: A subsample of 30 caregivers was randomly selected using a random number table and retested after a two-week interval. Data were collected at two separate time points. We then computed the intraclass correlation coefficient (ICC) for both the overall scale and its sub-dimensions to assess stability. The ICC ≥ 0.70 was considered to indicate adequate stability (23).

#### 2.3.3 Validity assessment

Structural Validity: Factor analysis, including exploratory factor analysis (EFA) and confirmatory factor analysis (CFA), was used to examine structural validity. For EFA, the Kaiser–Meyer–Olkin (KMO) measure and Bartlett’s test of sphericity were first examined; a KMO value >0.6 and a significant Bartlett’s test (*p* < 0.05) were required to proceed (29). We performed a principal component analysis using varimax rotation, retaining factors with eigenvalues exceeding 1.0. Items with factor loadings <0.40 were removed (30), and a cumulative variance contribution rate >50% was considered indicative of good structural validity. Based on the EFA results, CFA was performed using maximum likelihood estimation. Model fit was assessed using the chi-square/degrees of freedom ratio (*χ^2^/df*), root mean square error of approximation (RMSEA), root mean square residual (RMR), goodness-of-fit index (GFI), comparative fit index (CFI), Tucker–Lewis index (TLI), and incremental fit index (IFI). Convergent validity was assessed using factor loadings, composite reliability (CR), and average variance extracted (AVE), while discriminant validity was evaluated based on correlations between dimensions. Content validity was assessed using the I-CVI and S-CVI, with acceptance criteria set at I-CVI ≥ 0.78 and S-CVI ≥ 0.90. Expert authority was quantified using the Cr, with Cr ≥ 0.70 considered acceptable.

## 3 Results

### 3.1 Linguistic validation and content validity

The linguistic validation process resulted in a Chinese version of the FCCT-CI that achieved semantic and conceptual equivalence with the original English version. During the translation process, several items were refined to enhance cultural and linguistic appropriateness. First, the scale title was shortened from “慢性病患者家庭照顾者沟通模式评估量表” (Chronic Disease Family Caregiver Communication Assessment Scale) to “慢性病患者家庭照顾者沟通模式量表” (Chronic Disease Family Caregiver Communication Scale) to avoid redundancy, as the term “评估” (assessment) was deemed unnecessary for a standardized measurement tool. Second, the phrase “my loved” was consistently translated as “患者” (patient) across all items to reflect the Chinese caregiving context. Third, the phrase “After a medical appointment” was translated as “患者就诊后” (after the patient’s medical visit) rather than the literal “在医疗预约之后” (after the medical appointment) to improve comprehension among family caregivers. Fourth, “without spending too much time” was rendered as “很快达成共识” (quickly reach a consensus) rather than “没有花费太多时间” (did not spend too much time) to better capture the natural Chinese expression of consensus-building. The two forward translations showed high consistency, and only minor wording discrepancies were resolved through team discussion. Content validity was evaluated by a panel of 10 experts. The S-CVI was 0.94, and I-CVIs ranged from 0.80 to 1.00, indicating excellent content validity. The Cr was 0.92, exceeding the recommended threshold of 0.70, indicating that the expert consultation results were highly reliable. Detailed expert opinions and the corresponding item modifications are presented (Table 1).

**Table 1:** Expert opinions and revision results.

Original translation version	Revision comments	Revised version
1.我主动与家人谈论患者的病情, 包括线上联络与短信的方式。	“线上联络与短信”改为“以线上和短信的方式”以更贴合中文表达	1.我主动与家人谈论患者的病情, 包括以线上和发短信的方式。
4.在我们的家庭中, 我们会谈论患者的未来照顾计划。	①“谈论”改为“讨论”以更符合中文表达习惯	4.我会与家人讨论对患者未来的照顾计划。
	②“在我们的家庭中”一词多余, 建议去掉	
5.我的家人讨论如果治疗不起作用, 可能会发生什么。	①“治疗不起作用”改为“治疗无效”	5.我的家人会一起讨论如果患者治疗无效, 我们该如何应对。
	②结合量表语义及背景建议将“可能会发生什么”翻译为“我们该如何应对”	
6.我需要在照护方面得到家人的认可。	“认可”翻译为“支持”这能够体现照顾者在照护过程中希望得到家人的积极帮助和鼓励	6.我需要在照护方面得到家人的支持。
8.在接受家人关于医疗照护的信任时, 我感到有压力。	“信任”改为“观念或想法”以使语句更连贯。	8.在接受家人关于医疗照护的观念时我感到有压力。
9.我的家人隐瞒了他们对我照护质量的意见。	①“意见”可能不够准确, 需重新翻译	9.我的家人对我的照护质量保持沉默。
	②“隐瞒了他们意见”改为“对我的照护质量保持沉默”以更贴合中文表达	
10.我的家人试图表现出我们所爱的人好像没有生病一样。	“试图表现出”改为“尽量表现出”以更符合中文表达	10.我的家人尽量表现出患者好像没有生病一样。

### 3.2 Participant characteristics

The total analyzed sample consisted of 319 older adult patients with chronic diseases and their 319 family caregivers (Table 2). Among the caregivers, women accounted for the majority (63.3%), and the largest age group was 45–59 years (43.9%). Regarding the patients, 63% were male, 43.3% were aged 60–69 years, and 46.7% had moderate dependence on others for self-care (Table 3).

**Table 2:** General information of family caregivers (*n* = 319).

Item	Category	Frequency	Percentage (%)
Gender	Male	117	36.7
	Female	202	63.3
Age (years)	18–44	86	27.0
	45–59	140	43.9
	≥60	93	29.2
Residence	Urban	173	54.2
	Rural	146	45.8
Education level	Primary school or below	81	25.4
	Junior high school	107	33.5
	Senior high school/Technical school	100	31.3
	College/Undergraduate or above	31	9.7
Relationship with Patient	Spouse	106	33.2
	Child	192	60.2
	Other	21	6.6
Employment status	Employed	175	54.9
	Unemployed	29	9.1
	Retired	115	36.1
Number of co-caregivers	0	78	24.5
	1	129	40.4
	2	78	24.5
	≥3	34	10.7
Duration of caregiving (years)	<1	89	27.9
	1–5	126	39.5
	6–10	65	20.4
	>10	39	12.2
Daily caregiving time (hours)	≤4	165	51.7
	5–8	45	14.1
	≥9	109	34.2

**Table 3:** General information of patients with chronic diseases (*n* = 319).

Item	Category	Frequency	Percentage (%)
Gender	Male	201	63.0
	Female	118	37.0
Age (years)	60–69	138	43.3
	70–79	121	37.9
	≥80	60	18.8
Marital status	Married	203	63.6
	Unmarried	8	2.5
	Divorced	15	4.7
	Widowed	93	29.2
Education level	Primary school or below	158	49.5
	Junior high school	87	27.3
	Senior high school/Technical school	52	16.3
	College/Undergraduate or above	22	6.9
Medical insurance payment method	Self-pay	5	1.6
	Urban employee medical insurance	98	30.7
	Urban and rural resident medical insurance	209	65.5
	Publicly funded medical care	7	2.2
Patient’s self-care ability	Mild dependence	43	13.5
	Moderate dependence	149	46.7
	Severe dependence	127	39.8

### 3.3 Item analysis

We utilized the entire sample (*N* = 319) to assess the discriminative ability of the items. The analysis revealed that the correlation coefficients between individual items and the total score fell within the range of 0.576 to 0.719 (*p* < 0.01). Importantly, all values surpassed the recommended cutoff of 0.40. The Critical Ratio (CR) values, comparing the highest 27% and lowest 27% scoring groups, ranged from 11.921 to 17.000(*p* < 0.001), indicating strong discriminative power.

### 3.4 Structural validity

#### 3.4.1 Exploratory factor analysis

**Figure 1:**
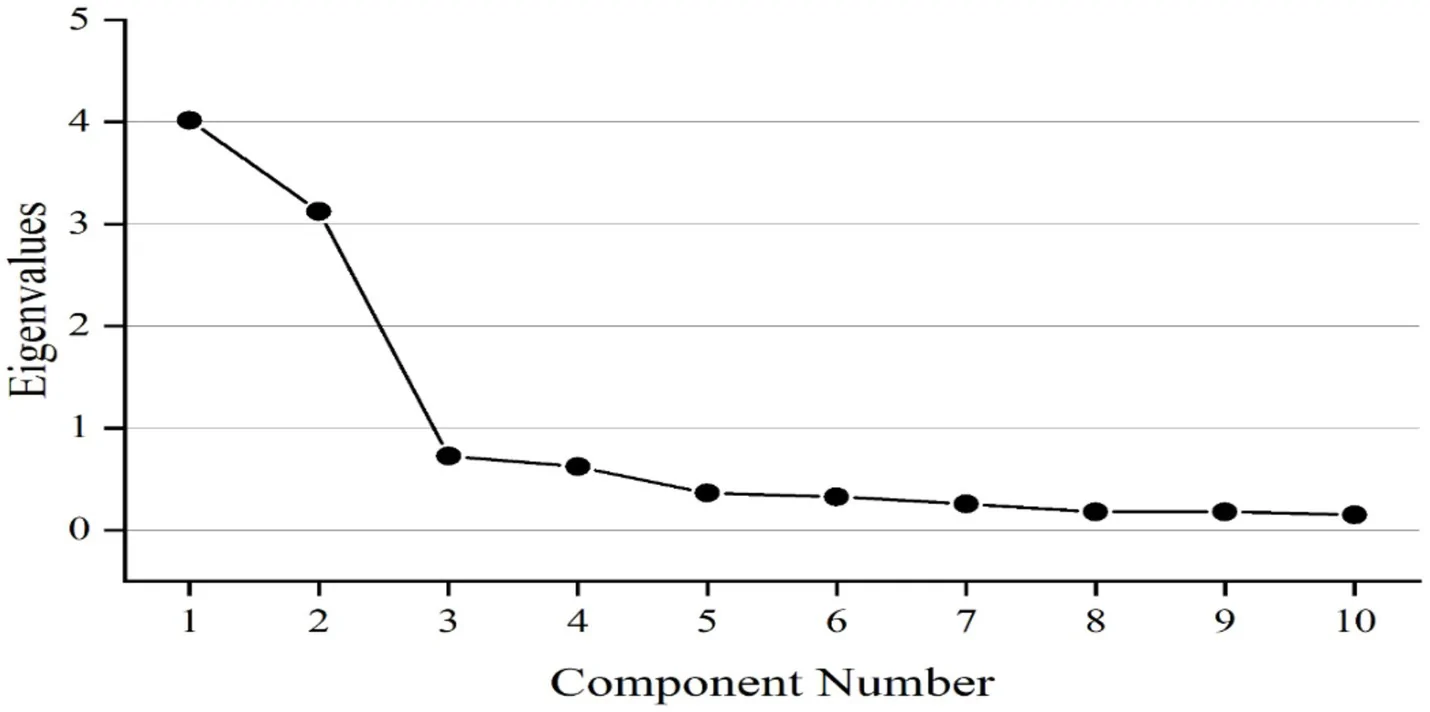
The scree plot of the FCCT-CI.

Exploratory Factor Analysis (EFA) was performed using the data from Subsample A (*N* = 110). First, we assessed the suitability of the data for this analysis. The Kaiser-Meyer-Olkin (KMO) value was calculated to be 0.80. Furthermore, Bartlett’s test of sphericity showed statistical significance (*χ^2^* = 722.66, *df* = 45, *p* < 0.001), which confirmed that the dataset was appropriate for factor analysis. We then applied Principal Component Analysis (PCA) with Varimax rotation to extract the factors. The scree plot shows an inflection point after the second factor and then plateaus (Figure 1). Two distinct factors emerged with eigenvalues exceeding 1.0, specifically 4.008 and 3.139. Cumulatively, these factors explained 71.46% of the total variance. Factor 1 was identified as Conversation Orientation, while Factor 2 was labeled Conformity Orientation; each dimension comprised 5 items. The rotated factor loadings ranged between 0.782 and 0.879 (Table 4), all above 0.40, with no significant cross-loadings identified. The two factors were moderately correlated (*r* = 0.197), indicating that they are related but represent distinct dimensions of family communication patterns.

**Table 4:** Factor loadings from EFA.

Dimension	Item	Factor loading	
		Factor 1	Factor 2
Conversation	Q1	–	0.822
	Q2	–	0.835
	Q3	–	0.849
	Q4	–	0.849
	Q5	–	0.782
Conformity	Q6	0.796	–
	Q7	0.863	–
	Q8	0.867	–
	Q9	0.879	–
	Q10	0.814	–

“–” indicates factor loadings <0.40.

#### 3.4.2 Confirmatory factor analysis

Confirmatory Factor Analysis (CFA) was conducted using the data from Subsample B (*N* = 209). The primary aim of this step was to verify the stability of the factor structure.

The initial model did not achieve satisfactory fit to the data. Model modification was guided by modification indices (MI), with MI > 5 considered indicative of necessary residual covariance adjustments (23). Seven covariance paths were added between residuals of items within the same dimension. All covariances were theoretically justified and consistent with the original two-factor structure. After these modifications, the revised model demonstrated adequate fit to the data, as shown by the following fit indices: *χ^2^*/*df* = 1.707, RMSEA = 0.058, RMR = 0.098, GFI = 0.959, CFI = 0.988, IFI = 0.988, and TLI = 0.979.

**Figure 2:**
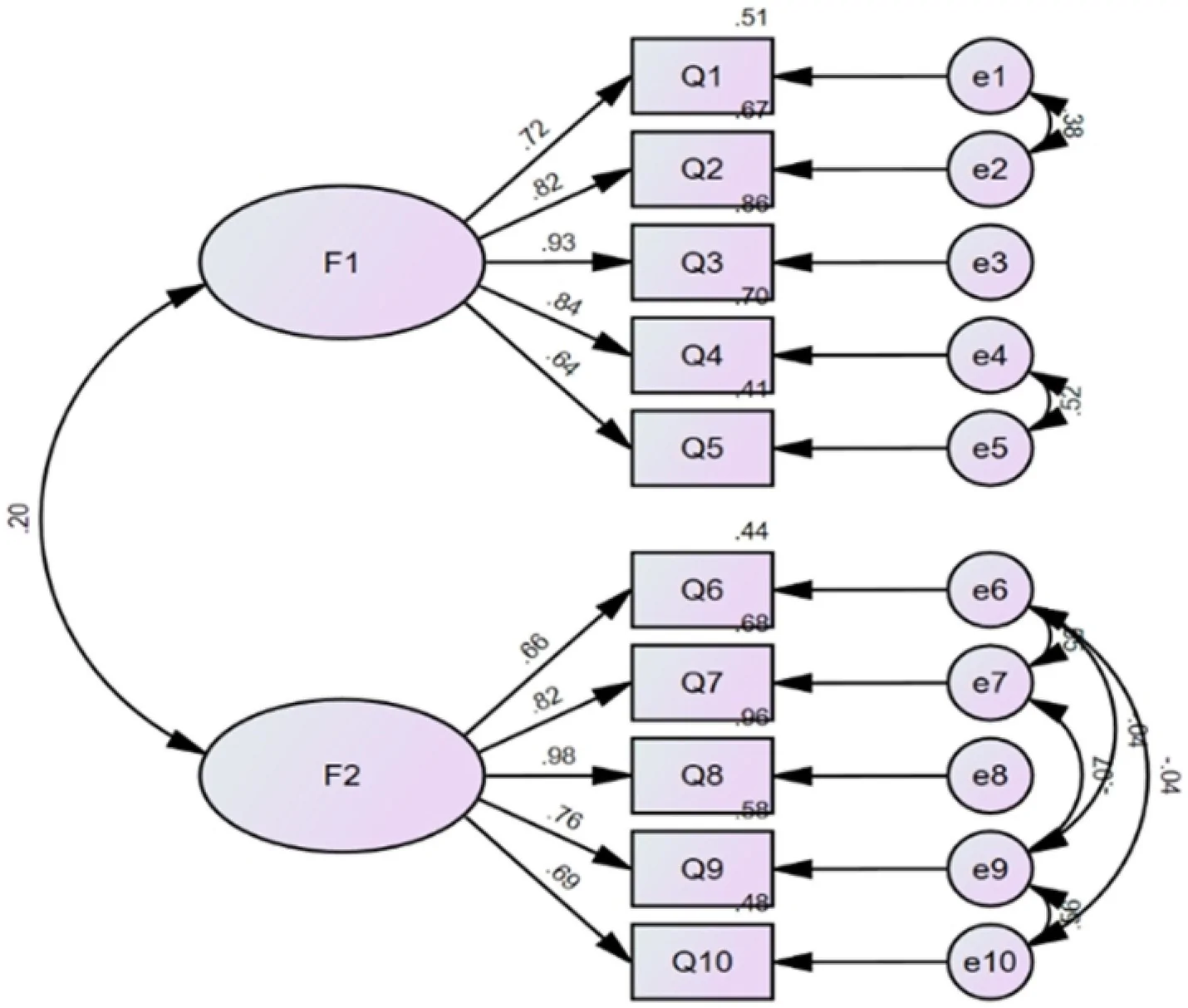
Standardized two-factor model of the Chinese version of the FCCT-CI. The numbers near the 1-headed arrows are factor loading coefficients; the numbers near the 2-headed arrows are correlation coefficients; the numbers aiming to the items through a 1-headed arrow are residual variances. F1, conversation; F2, conformity; Q1, item 1.

The CFA confirmed a stable two-factor structure comprising Conversation and Conformity dimensions, with each dimension containing five items. All standardized factor loadings were statistically significant (*p* < 0.001). For the Conversation dimension, loadings ranged from 0.638 to 0.929; for the Conformity dimension, loadings ranged from 0.663 to 0.978 (Figure 2). The standard errors ranged from 0.076 to 0.159, indicating good model stability.

#### 3.4.3 Convergent and discriminant validity

Convergent validity was supported by the Average Variance Extracted (AVE) and Composite Reliability (CR). The AVE values for the two factors were 0.630 and 0.625, respectively (both > 0.50). The CR values were 0.893 and 0.891 (both > 0.70). Discriminant validity was established as the square roots of the AVE (0.794 and 0.791) were both greater than the inter-factor correlation coefficient (0.197).

### 3.5 Reliability

The scale demonstrated high reliability. The Cronbach’s *α* coefficient for the total scale was 0.846, with 0.901 and 0.906 for the Conversation and Conformity subscales, respectively. The split-half reliability was 0.943. Test–retest reliability (ICC) over a two-week interval (*N* = 30) was 0.920 for the total scale (*p* < 0.01).

## 4 Discussion

### 4.1 Psychometric robustness and cross-cultural applicability

The present study rigorously followed international guidelines to translate and validate the FCCT-CI for the Chinese population. The results indicate that the Chinese version demonstrates satisfactory psychometric properties and maintains conceptual equivalence with the original English version developed by Wittenberg et al. (18).

A Cronbach’s *α* of 0.846 was measured in this study, demonstrating excellent internal consistency. This value is highly consistent with the original report (α range: 0.74–0.86) and even outperforms it in aspects such as test–retest reliability (ICC = 0.920). Structural validity was comprehensively assessed using both exploratory and confirmatory factor analyses, and the results confirmed two stable factors (Conversation and Conformity) consistent with the original dimensional framework. These findings suggest that despite significant linguistic and cultural differences between Western and Eastern societies, the core constructs of family communication regarding chronic illness are cross-culturally translatable and highly relevant to the Chinese context. Specifically, the tension between open communication and adherence to family norms constitutes a universal theme in caregiving (31, 32).

### 4.2 Cultural interpretation: collectivism and family dynamics

According to Family Communication Patterns Theory, family communication consists of two core dimensions: conversation orientation, which emphasizes encouraging family members to freely express their personal thoughts, and conformity orientation, which emphasizes the importance of family harmony and social relationships, minimizing conflict by expecting family members to accept shared values. The confirmation of the Conformity dimension is particularly meaningful and warrants deep interpretation within the Chinese cultural context. Unlike Western individualistic cultures, where “conformity” often implies the suppression of personal autonomy and is typically viewed as a dysfunctional communication pattern, Chinese caregiving is deeply embedded in a collectivist framework influenced by Confucianism (33, 34). In this context, the meaning of conformity is considerably more nuanced, as adherence to family norms is not merely compliance but an active expression of moral responsibility.

Two specific cultural values likely contributed to the distinct performance of the Conformity dimension in this study. First is the concept of Filial Piety, or Xiao. In Chinese families, adult children often view caregiving not just as a duty but as a moral imperative; this cultural logic operates through the internalization of family expectations. Younger family members align their decisions with elders not simply out of obligation, but because doing so is perceived as an enactment of filial virtue, a process that transforms conformity from passive submission into active moral engagement (35, 36). This hierarchy often requires younger family members to align their decisions with elders to show respect, which leads to high conformity scores regarding caregiving plans (33, 34). Consequently, items in the Conformity dimension all yielded high factor loadings in this study. Notably, Q9 (“My family talks about what might happen if treatment does not work”) loaded at 0.879, and Q10 (“My family tries to act as though my loved is not ill”) loaded at 0.814. The family discussion about potential treatment failure reflected in Q9 appears to be interpreted by Chinese caregivers as a prudent consideration of family responsibility rather than a restriction on personal expression. Meanwhile, Q10 directly corresponds to the cultural logic of protective buffering—namely, maintaining the appearance of normalcy by avoiding discussions of illness severity to preserve family stability.

The second factor is Family Harmony, known as He. The preservation of harmony is paramount in Chinese family life. Within this cultural logic, harmony is maintained through the subordination of individual preferences to collective family interests, thereby preserving relational stability during health crises. This cultural orientation often manifests as “protective buffering,” a phenomenon where family members hide bad news, suppress their own distress, or avoid discussing the severity of the illness to protect the patient from psychological harm (37, 38). Consequently, items in the Conformity dimension, such as hiding opinions or acting as if the patient is not ill, resonate strongly with Chinese caregivers. This is particularly evident in Q10 (“My family tries to act as though my loved is not ill”), which loaded at 0.814 in the present study, indicating that this item strongly captures the shared cultural experience of protective buffering. Ultimately, this behavior should not be viewed merely as poor communication, but rather as a culturally specific strategy to maintain family stability during a health crisis (39).

### 4.3 Comparison with existing instruments

The validation of the FCCT-CI fills a significant gap in the current landscape of measurement tools available in China. Previous instruments have shown limitations in scope. For instance, the Family Avoidance of Communication about Cancer Scale focuses narrowly on avoidance behaviors and is disease-specific (40). This limits its utility for general chronic conditions like hypertension or diabetes. Similarly, the Caregiver-Centered Communication Questionnaire primarily assesses the interaction between caregivers and healthcare providers rather than the internal dynamics among family members (41, 42).

In addition to these disease-specific or provider-focused tools, several generic caregiver scales have recently been validated in the Chinese context, providing useful benchmarks for comparison. Chen et al. (43) cross-culturally adapted the Caregiver Contribution to Self-Care Chronic Illness Inventory among 301 Chinese caregivers and reported Cronbach‘s *α* values ranging from 0.792 to 0.870 across its three subscales, with test–retest ICCs between 0.728 and 0.783. However, the CFA fit indices for the maintenance and management subscales were suboptimal, with CFI values of 0.822 and 0.939 respectively, suggesting that the factor structure of this instrument may require further refinement in the Chinese population. Lv et al. (44) validated the Caregiver Self-Efficacy in Contributing to Patient Self-Care Scale among 406 caregivers from four tertiary hospitals in Tianjin, demonstrating strong internal consistency with a Cronbach’s *α* of 0.935 and good test–retest reliability with ICCs ranging from 0.784 to 0.829. While the CSE-CSC offers a valuable measure of caregiver self-efficacy, its focus is on caregivers ‘confidence in assisting with patient self-care tasks, which is conceptually distinct from the relational and communicative dynamics captured by the FCCT-CI. Notably, the FCCT-CI Cronbach‘s α of 0.846 is comparable to that of the C-CC-SC-CII and lower than that of the CSE-CSC. This difference is likely attributable to the multidimensional nature of communication patterns versus the relatively unidimensional construct of self-efficacy. Furthermore, the FCCT-CI demonstrated superior test–retest reliability with an ICC of 0.920 compared to both instruments, suggesting that family communication patterns represent a more stable, trait-like construct than self-care contributions or self-efficacy beliefs. The CFA fit indices for the FCCT-CI also compared favorably to those reported for the CSE-CSC, with CFI values of 0.988 versus 0.973, RMSEA values of 0.058 versus 0.082, and TLI values of 0.979 versus 0.960, supporting the robustness of the two-factor theoretical framework derived from Family Communication Patterns Theory.

In terms of cultural adaptation strategies, both the C-CC-SC-CII and CSE-CSC made specific modifications to enhance cultural relevance. Chen et al. (43) replaced the abstract term “healthcare provider” with “doctors or nurses” to improve comprehension among Chinese caregivers. Lv et al. (44) refined “the person you care for” to “patient” for clarity and explicitly specified that “treatment plan” refers to plans prescribed by medical professionals, reflecting the reality that Chinese caregivers sometimes seek health advice from unofficial sources. These modifications, along with our own adjustments such as translating “trust” as “观念或想法” and clarifying communication modalities, share a common logic. Effective cross-cultural adaptation requires transforming abstract or Western-centric concepts into concrete, culturally accessible cognitive units, rather than relying on literal translation. Collectively, these comparison findings affirm that the Chinese FCCT-CI holds a distinct position among available caregiver assessment tools. It is the first theory-driven, reliable, and valid measure specifically designed to capture intra-family communication patterns in the context of chronic illness caregiving in China.

In contrast, the FCCT-CI captures the complexity of intra-family interactions by measuring both the frequency of dialog in the Conversation dimension and the pressure to agree in the Conformity dimension. This two-factor framework captures a more complete picture of familial interactions. It effectively distinguishes between families who communicate openly and those who suppress information to maintain a facade of normalcy (44, 45).

### 4.4 Clinical implications

There is an accelerated aging process within its population in China, which is accompanied by a growing load of chronic illnesses. Consequently, the contribution made by family caregivers has assumed a position of paramount importance. However, studies indicate that Chinese caregivers often suffer in silence and remain unaware that their communication patterns may contribute to their burden (46, 47). The validated FCCT-CI offers significant clinical utility in two key areas.

First, the brevity of the scale makes it an ideal screening tool for busy clinical settings. Nurses and social workers can use it during discharge planning or community health visits to identify families with maladaptive patterns. For example, caregivers with a Protective style, characterized by low conversation and high conformity, are at higher risk for burnout and patient isolation.

Second, understanding a family’s communication typology allows for more precise interventions. For families exhibiting high conformity, clinicians should not simply force open communication, as this may violate their cultural norms. Instead, interventions should aim to create a safe space for caregivers to express their needs while respecting their desire for harmony. This could be achieved by reframing communication as a way to enhance the quality of care for the patient (48, 49).

### 4.5 Limitations and future directions

Several limitations of this study should be acknowledged. First, participants were recruited via convenience sampling from a single tertiary hospital in Henan Province. As economic status and education levels vary significantly across China, findings may lack broad representativeness to other areas. This limitation is shared with other recent Chinese validation studies of caregiver scales, such as Chen et al. (Henan, single-province) and Lv et al. (Tianjin, four hospitals within one city). However, Henan Province, as one of China‘s most populous regions with a typical urban–rural dual structure 54.2% urban and 45.8% rural in our sample does provide coverage of major caregiving scenarios to some extent. Nonetheless, multi-center validation across diverse socioeconomic regions remains a priority for future research. Additionally, the data were derived entirely from self-reports. This method may introduce social desirability bias, especially given the cultural tendency to present a harmonious family image to outsiders. Caregivers may over-report socially desirable items while underreporting conflict-related ones. This could systematically inflate Conversation orientation scores and underestimate the true burden reflected in Conformity orientation. Third, although our EFA sample (*n* = 110) met the minimum requirement of 10 subjects per item, the relatively modest sample size for factor analysis may affect the robustness of the factor structure. We mitigated this by validating the structure with an independent CFA sample (*n* = 209), but cross-validation with larger samples is needed in future studies.

In addition to these methodological considerations, the cultural boundaries of the adapted scale deserve critical reflection. While the FCCT-CI demonstrated good psychometric properties in our sample, the meaning of “conformity” may differ fundamentally between Western and Chinese contexts. In Western settings, conformity is typically understood as submission to family authority; in Chinese caregiving, it may also reflect filial piety and a deliberate strategy to maintain family harmony through protective buffering. This does not compromise the scale’s validity within China, but it does caution against direct cross-cultural comparisons of raw scores without further equivalence testing. Future cross-national studies should conduct measurement invariance analyses to quantify how cultural context shapes item interpretation and response patterns.

Looking forward, four directions are particularly important. First, multi-center studies covering eastern, central, and western regions of China are needed to confirm the scale’s generalizability. Second, longitudinal designs should examine how caregiver communication patterns evolve as patients progress from diagnosis through advanced stages to end-of-life care. Third, future research could examine the scale’s performance across different caregiver subgroups, such as urban versus rural, or spousal versus adult-child caregivers, to assess its measurement invariance across these populations. Fourth, future studies could employ network analysis as a supplementary approach to explore the complex interplay and centrality of specific communication items (50, 51), offering a complementary perspective to traditional factor analysis.

At the policy level, the FCCT-CI can serve as a practical screening tool in community health services. Routine use of the scale could help identify families at risk for maladaptive communication patterns and enable targeted interventions. Specifically, the scale could be integrated into the older adult health management module of China’s Basic Public Health Services, with assessment results informing the allocation of psychological support resources. Given the growing burden of chronic disease in China’s aging population, systematic assessment of family communication has the potential to improve both caregiver wellbeing and patient outcomes, and deserves greater policy attention.

## 5 Conclusion

This study strictly followed the procedures for introducing foreign scales, translated the FCCT-CI into Chinese, and conducted reliability and validity tests. The structural composition of the Chinese FCCT-CI aligns perfectly with the original instrument. It is comprised of 10 specific items that are distributed across two distinct dimensions. In terms of usability, the scale is characterized by its brevity and is easy for participants to comprehend. Furthermore, it demonstrated satisfactory psychometric properties, specifically regarding reliability and validity. Consequently, the Chinese version of this scale serves as a valuable instrument. It can be effectively utilized to evaluate the communication patterns of family caregivers providing care to older adults with chronic conditions in China.

## Data Availability

The raw data supporting the conclusions of this article will be made available by the authors, without undue reservation.

## References

[1] World Health Organization. Noncommunicable Diseases. Available online at: https://www.who.int/news-room/fact-sheets/detail/noncommunicable-diseases (Accessed January 1, 2026).

[2] National Health Commission of the People's Republic of China; Office of the National Working Commission on Aging. Statistical Bulletin on the Development of National Aging Undertakings in 2022. Available online at: https://www.gov.cn/lianbo/bumen/202312/content_6920261.htm (Accessed November 15, 2025).

[3] National Health Commission of the People's Republic of China. Analysis Report of the Fifth National Health Services Survey 2013. Available online at: https://www.nhc.gov.cn/ewebeditor/uploadfile/2016/10/20161026163512679.pdf (Accessed December 3, 2025).

[4] Zhou M Wang H Zeng X Yin P Zhu J Chen W . Mortality, morbidity, and risk factors in China and its provinces, 1990–2017: a systematic analysis for the global burden of disease study 2017. Lancet. (2019) 394:1145–58. doi: 10.1016/S0140-6736(19)30427-1, 31248666 PMC6891889

[5] Li Y Wang Y Luo Q Wang A Yu H. Analysis on supporting service needs and influencing factors of elderly chronic family caregivers. Soft Sci Health. (2019) 33:79–83. doi: 10.3969/j.issn.1003-2800.2019.10.018

[6] Zhong X Chen S. Analysis of the burden and influencing factors of family caregivers for elderly cognitive impairment patients. Qingdao Med J. (2023) 55:255–7. doi: 10.3969/j.issn.1006-5571.2023.04.005

[7] Meng H Lu H Lv W Huang J Xu Y Zhang L. Correlation analysis between care burden and resilience of primary caregivers of older adult stroke patients. Chin Gen Pract Nurs. (2023) 21:1170–3. doi: 10.12104/j.issn.1674-4748.2023.09.004

[8] Waldenburger N Steinecke M Peters L Jünemann F Bara C Zimmermann T. Depression, anxiety, fear of progression, and emotional arousal in couples after left ventricular assist device implantation. ESC Heart Fail. (2020) 7:3022–8. doi: 10.1002/ehf2.12927, 32725771 PMC7524127

[9] Kedia SK Collins A Dillon PJ Akkus C Ward KD Jackson BM. Psychosocial interventions for informal caregivers of lung cancer patients: a systematic review. Psycho-Oncology. (2020) 29:251–62. doi: 10.1002/pon.5271, 31701588

[10] He X Chen F Yuan J Du X. Status quo and influencing factors of family resilience among family caregivers of the disabled elderly. Chin Evid Based Nurs. (2023) 9:3193–8. doi: 10.12102/j.issn.2095-8668.2023.17.029

[11] Han J Tang R Zhang Z. Why do Chinese patients avoid health information? The role of caregiver burden perception and self-stigma. Patient Educ Couns. (2025) 140:109275. doi: 10.1016/j.pec.2025.109275, 40729980

[12] Wang Y Zhang Y Hong Y Zeng P Hu Z Xu X . Advance directives and end-of-life care: knowledge and preferences of patients with brain tumours from Anhui, China. BMC Cancer. (2021) 21:25. doi: 10.1186/s12885-020-07775-4, 33402101 PMC7786498

[13] Liang L Li W. Effects of gender on emotional interactions in family elderly care. Collect Women’s Stud. (2024) 1:13–27.

[14] Siminoff LA Zyzanski SJ Rose JH Zhang AY. The cancer communication assessment tool for patients and families (CCAT-PF): a new measure. Psycho-Oncology. (2008) 17:1216–24. doi: 10.1002/pon.1350, 18504807 PMC2830149

[15] Zhang Y Xie Z Li Y Li P. Translation and validation of the family avoidance of communication about Cancer scale. J Nurs Sci. (2022) 37:9–12. doi: 10.3870/j.issn.1001-4152.2022.07.009

[16] Olthof-Nefkens MWLJ Derksen EWC de Swart BJM Nijhuis-van der Sanden MWG Kalf JG. Development of the experienced communication in dementia questionnaire: a qualitative study. Inquiry. (2021) 58:469580211028181. doi: 10.1177/00469580211028181 34167366 PMC8246470

[17] Demiris G DeKeyser Ganz F Han CJ Ganz FDK Pike K Oliver DP . Design and preliminary testing of the caregiver-centered communication questionnaire (CCCQ). J Palliat Care. (2020) 35:154–60. doi: 10.1177/0825859719887239, 31696787 PMC7202953

[18] Wittenberg E Bevan JL Goldsmith JV. Assessing family caregiver communication in chronic illness: validation of the FCCT-CI. Am J Hosp Palliat Med. (2023) 40:500–7. doi: 10.1177/10499091221106694, 35653264

[19] Koerner AF Fitzpatrick MA. Toward a theory of family communication. Commun Theory. (2002) 12:70–91. doi: 10.1111/j.1468-2885.2002.tb00260.x

[20] Tan M Tang S Huang C Xiao J Ding J. Summary of evidence to facilitate the implementation of advance care planning among advanced cancer patients. J Cent South Univ Med Sci. (2024) 49:135–44. doi: 10.11817/j.issn.1672-7347.2024.230291 PMC11017018 38615175

[21] Muñiz J Elosua P Hambleton RK. International test commission guidelines for test translation and adaptation: second edition. Psicothema. (2013) 25:151–7. doi: 10.7334/psicothema2013.24, 23628527

[22] Jones PS Lee JW Phillips LR Zhang XE Jaceldo KB. An adaptation of Brislin's translation model for cross-cultural research. Nurs Res. (2001) 50:300–4. doi: 10.1097/00006199-200109000-00008, 11570715

[23] Wu M. Structural Equation Modeling: Operation and Application of AMOS. Chongqing, China: Chongqing University Press (2018).

[24] How to test content validity. J Nurs Train. (2010) 25:37–9. doi: 10.16821/j.cnki.hsjx.2010.01.017

[25] Beaton DE Bombardier C Guillemin F Ferraz MB. Guidelines for the process of cross-cultural adaptation of self-report measures. Spine. (2000) 25:3186–91. doi: 10.1097/00007632-200012150-00014, 11124735

[26] Mahoney FI Barthel DW. Functional evaluation: the Barthel index. Md State Med J. (1965) 14:61–5. 14258950

[27] Li X Chen M. Design and application of the modified Barthel index rating scale. Chin Nurs Res. (2015) 29:1657–8. doi: 10.3969/j.issn.1009-6493.2015.13.044

[28] Hou D Zhang Y Wu J Li Y An Z. Study on reliability and validity of Chinese version of Barthel index. Clin Focus. (2012) 27:219–21.

[29] Wu ML. Questionnaire Statistical Analysis in Practice: SPSS Operation and Application. Chongqing, China: Chongqing University Press (2010).

[30] Shi J Mo X Sun Z. Content validity index in scale development. J Cent South Univ Med Sci. (2012) 37:152–5. doi: 10.3969/j.issn.1672-7347.2012.02.007 22561427

[31] Ruan T Huang Y Zhang L. Cross-cultural adaptation of the family coping questionnaire and psychometric evaluation among caregivers of schizophrenic patients in China. BMC Nurs. (2025) 24:853. doi: 10.1186/s12912-025-03534-7, 40618043 PMC12229010

[32] Xu RH Xu Y Zhao M Liu N Wang P Liang X . Psychometric validation and cultural adaptation of the Chinese version of the CarerQol-7D instrument. Health Qual Life Outcomes. (2025) 23:49. doi: 10.1186/s12955-025-02379-7, 40346608 PMC12065290

[33] Jia H Zhang J Su W Wei Z Yang L Wang Y. The impact of caregiver burden on sense of coherence in Chinese family caregivers of people with dementia: the mediating effect of family resilience. BMC Psychol. (2025) 13:369. doi: 10.1186/s40359-025-02678-0, 40217520 PMC11987476

[34] Garrido SC Teixeira S Mora-Lopez G Sampaio F. Factors associated with anxiety, stress, depression and burden among informal caregivers of patients with dementia: a cross-sectional study. BMC Nurs. (2025) 24:1384. doi: 10.1186/s12912-025-04014-8, 41219763 PMC12604331

[35] Xiao C Patrician PA Montgomery AP Wang Y Jablonski R Markaki A. Filial piety and older adult caregiving among Chinese and Chinese-American families in the United States: a concept analysis. BMC Nurs. (2024) 23:115. doi: 10.1186/s12912-024-01789-0, 38347512 PMC10863110

[36] Chang CC Hsu K Chen CM Huang SS Wu IC Hsu CC . Gender difference on the mediation effects of filial piety on the association between chronic obstructive pulmonary disease and depressive symptoms in older adults: a community-based study. PLoS One. (2024) 19:e0298360. doi: 10.1371/journal.pone.0298360, 38386662 PMC10883558

[37] Wang H Yue H Wang Z Lu M Feng D. The relationship between patient-family caregiver congruence/incongruence in acceptance of illness and family caregivers' anticipatory grief. Psychooncology. (2023) 32:751–9. doi: 10.1002/pon.6121, 36890764

[38] Li S Zhang L Fang Y. Does social support alleviate the caregiving burden of adult children? Evidence from Chinese long-term care insurance pilot program. J Aging Soc Policy. (2025) 37:359–74. doi: 10.1080/08959420.2024.2384178, 39422055

[39] Wang Y Cai C Zhu Y Shi W Cheng B Li C . Family burden and psychological distress among Chinese caregivers of elderly people with dementia: a moderated mediation model. BMC Nurs. (2024) 23:723. doi: 10.1186/s12912-024-02382-1 39379874 PMC11462764

[40] Qiao Y Duan P Wang X Yang L Zou Y Fei C. Analysis of current status and influencing factors of family avoidance of cancer communication in 173 patients with advanced cancer. J Nurs (China). (2023) 30:18–23. doi: 10.16460/j.issn1008-9969.2023.13.018

[41] Hu Y Xie J Wei X Guo D Yuan T Wang L . Application of cognitive interviewing in cross-cultural adjustment of caregiver-Centered communication questionnaire. Chin Nurs Res. (2023) 37:3163–6. doi: 10.12102/j.issn.1009-6493.2023.17.021

[42] Starr LT Bullock K Washington K Aryal S Parker Oliver D Demiris G. Anxiety, depression, quality of life, caregiver burden, and perceptions of caregiver-centered communication among black and white hospice family caregivers. J Palliat Med. (2022) 25:596–605. doi: 10.1089/jpm.2021.0302, 34793244 PMC8982115

[43] Chen DD Zhang H Cui N Tang L Shao J Wang X . Cross-cultural adaptation and validation of the caregiver contribution to self-care of chronic illness inventory in China: a cross-sectional study. BMJ Open. (2021) 11:e048875. doi: 10.1136/bmjopen-2021-048875, 34493514 PMC8424873

[44] Lv Q Zhang X Wang Y Xu X Zang X. Cross-cultural adaptation and validation of the caregiver self-efficacy in contributing to patient self-care scale in China. BMC Public Health. (2024) 24:1977. doi: 10.1186/s12889-024-19534-2, 39049013 PMC11267960

[45] Xu X Wang Y Meng J Cao W Liu Y. Reliability and validity of the Chinese version of the family resilience inventory. Front Psychol. (2025) 16:1456132. doi: 10.3389/fpsyg.2025.1456132, 40486890 PMC12142059

[46] Hu H Hu X Xu Y. Caring load and family caregivers’ burden in China: the mediating effects of social support and social exclusion. Front Public Health. (2023) 11:1194774. doi: 10.3389/fpubh.2023.1194774, 37809000 PMC10556706

[47] Yang Z Sun F Zhao L . Self-efficacy and well-being in the association between caregiver burden and sleep quality among caregivers of elderly patients having multiple chronic conditions in rural China: a serial multiple mediation analysis. BMC Nurs. (2023) 22:424. doi: 10.1186/s12912-023-01504-5 37957638 PMC10641968

[48] Wang L Li Y Zhao R Li H. Development and psychometric evaluation of the needs assessment questionnaire for family caregivers of end-of-life older adults in home hospice care. Sci Rep. (2025) 15:13146. doi: 10.1038/s41598-025-97475-5, 40240452 PMC12003740

[49] Wang L Li Y Zhao R Li J Gong X Li H. Influencing factors of home hospice care needs of family caregivers of the older adult with chronic diseases at the end of life in China: a cross-sectional study. Front Public Health. (2024) 12:1348285. doi: 10.3389/fpubh.2024.1348285, 38756894 PMC11098011

[50] Zhang Z Lin R Qiu A Wu H Wu S Zhang L . Application of DASS-21 in Chinese students: invariance testing and network analysis. BMC Public Health. (2024) 24:2934. doi: 10.1186/s12889-024-20123-6, 39443919 PMC11515758

[51] Song Z Ye J Song X Zhang Z Xu P Shen H. Development and psychometric properties of work information anxiety questionnaire. Psychol Res Behav Manag. (2023) 16:4629–46. doi: 10.2147/PRBM.S435356, 38024659 PMC10644875

